# Populations of Rice Grain Bug, *Paraeuscosmetus pallicomis*, (Hemiptera: Lygaeidae) in Weed-free Paddy Field, Weedy Paddy Field and Paddy Dykes

**DOI:** 10.21315/tlsr2017.28.2.1

**Published:** 2017-07-31

**Authors:** Tamrin Abdullah, Andi Nasruddin, Nurariaty Agus

**Affiliations:** Department of Pests and Diseases, Faculty of Agriculture, Hasanuddin University, Makassar 90245, Indonesia

**Keywords:** *Paraeuscosmetus pallicomis*, Weedy, Weed-free, Dyke

## Abstract

Research on the populations of rice grain bug *Paraeuscosmetus pallicomis* Dallas (Hemiptera: Lygaeidae) in paddy field ecosystems was performed with the aim to determine the populations of rice grain bug in weed-free paddy field, weedy paddy field, and paddy dykes. Experiment was carried out in the village of Paccellekang in the district of Patallasang of Gowa Regency in South Sulawesi, Indonesia. Observations were performed during the milky grain stage (85 days after planting), the mature grain stage (105 days after planting), and one day after harvest (115 days after transplanting). Results showed that 85 days after the transplanting, the populations of rice grain bug was significantly higher in the weedy paddy field compared to weed-free field and paddy dykes with total numbers of 1.75, 3.53, and 0.31 insects per 2 hills, respectively. Similarly, 105 days after the transplanting, 2.53, 5.53, and 0.11 insects per hill, respectively. However, one day after the harvest (115 days after transplanting) the number of insects in weed-free field decreased, while in the dykes increased, and the weedy plot still had the highest number of insects per 2 hills. Our results suggested that weeds played an important role in regulating the bug population by providing alternative shelter and foods for the insect.

## INTRODUCTION

The world population continues to rise steeply every year, this rapid growth must be balanced by a sufficient increase in food, clothing, and housing. Rice is the staple food for a majority of Indonesians, thus its availability must be preserved. National food security has experienced stagnation and is not yet adequate to fulfill the dietary needs of the country. In efforts to increase the production of rice, the influence of various biotic and abiotic factors plays a determining role. One such factor is plant-destroying organisms such as mice, rice white stemborers, brown planthoppers, rice tungro virus, and the attacks of new pests like the rice grain bug, *Paraeuscosmetus pallicomis* Dallas (Hemiptera: Lygaeidae) (Patta *et al*. 2009).

Rice grain bug was reported for the first time attacking rice in North Sulawesi Province, Indonesia (Sembel 1991, Pelealu 1991). The presence of the insect was later on detected in the Provinces of East Kalimantan and East Nusa Tenggara (Rauf & Lanya 2009) and currently the insect has been reported from all districts in South Sulawesi (Wibowo 2009).

The rice grain bug was first reported on rice in Mangkutana, the regency of East Luwu, but at that time it was not recognised as a rice pest. The insect was officially identified as a rice plant pest in 2009. Rice grain bug attacked an area of 397 hectares in Luwu, 37 hectares in East Luwu, 3 hectares in Palopo, and 171 hectares in North Luwu during the planting season of 2008 (Patta *et al*. 2009).

Reproduction and population increase of the rice grain bug were swift. It is estimated that hundreds of hectares of paddy fields in South Sulawesi are at risk of crop failure as a result of rice grain bug attacks (Pajung 2010). The insect was also reported to have affected crops in the regencies of Wajo, Gowa and Pinrang. In Wajo, the insect affected thousands of hectares of crops (Risnandi 2011). Pinrang was only just experiencing the arrival of this pest; first sightings were reported in the village of Labbakkang (Yahya 2011).

It is apparent that the presence of this pest over time is increasingly harmful to crop yield, and there is an immediate urgency to discover methods of controlling their spread in order to minimise further agricultural losses (Warouw *et al.* 1997).

Use of insecticides has thus far proven to be the fastest and most effective means of suppressing the population of rice grain bug, however precautions must be taken to prevent excessive use of such chemicals to avoid economic drawbacks and environmental pollution (Warouw *et al.* 1997).

In case of scarcity of primary target plants, most pests have alternative sources to feed from. For example, army cutworm prefers pinnate tansy mustard (*Descurainia pinnata*) over wheat; however, when wheat becomes scares the insect will switch to wheat and becomes pest (Capinera 2005). Therefore, the purpose of the current study was to determine the population rice grain bug in three different habitats, weed-free field, weedy field, and paddy dyke. Weed-free paddy field was a field in which weeds were mechanically removed weekly; weedy paddy field was a field where weed control was not applied; and paddy dykes were covered with weeds because herbages were allowed to grow throughout the research period.

## MATERIALS AND METHODS

This research was carried out in the village of Pacellekang in the district of Patallassang in Gowa, South Sulawesi. Treatments were arranged in a randomised complete block design with three replications. The treatments were (1) weed-free paddy field: weeds were mechanically removed from the field; (2) weedy paddy field: weed control was not performed on these plots; and (3) paddy dykes: herbage was allowed to grow throughout the research.

The fields for weedy and weed-free fields were tilled using a hoe and two-wheeled tractor (hand tractor). Seedling of rice cv IR-66 were transplanted from the nursery when the seedlings were 21 days old, with a planting space of 30 × 30 cm. Dead seedlings were replaced with rice seedlings of the same variety one week after the transplanting. Compound fertilizer (NPK) with a dose of 150 kg/ha was distributed evenly throughout the paddy fields. Pesticides were not applied during the study.

### Observation and Sampling

Observations were performed three times: (1) when the crops had reached the milk grain phase about 85 days after planting; (2) when the crops reached the mature grain stage at about 105 days after planting; and (3) one day after harvest (115 days after planting). Rice grain bug specimens were collected with a 12-volt dust vacuum. Two rice hills served as a single sample unit; each sample unit was placed in containment moments prior to specimen collection. The containment was made from a 0.5m × 0.5m × 0.9m wooden framework wrapped in gauze. Vacuuming was performed on the leaves and stem of the rice. For the third observation period (115 days after planting (one day after harvest), vacuuming was performed on the ratoon crops. Rice grain bug collection from the paddy dykes was also done using the dust vacuum and containment. Vacuuming was performed on the soil surface and all vegetation over a 0.3 m^2^ area, which is about the area occupied by two rice hills. There were eight sample units for each repetition; the experiment was performed three times for each treatment, resulting a total of 24 sample units for each treatment or 72 sample units per observation period.

For both weed-free and weedy paddy plots, four sample units were taken from the plot edges and four sample units from the plot centre. Those sample units taken from plot centre were at least 10 m from paddy dykes, and those sample units from plot edges were ± 1 m distance from the dykes. For the paddy dyke treatment, two sample units were taken from each dyke. Since each plot had four dykes, there were eight units per observation. It was necessary to enclose sample units and use a dust vacuum for specimen collection to ensure that all insects in a sample unit were gathered and rice grain bug population counts were accurate. All collected arthropods were placed into a collection bottle containing 70% alcohol. The arthropods were then brought to our laboratory to be sorted and counted.

Rice grain bugs were counted and categorised based on their stage of development: imago, large nymph, medium nymph, and small nymph. The specimens were morphologically identified under a microscope (Stemi DV4 stereo microscope, Carl Zeiss, Germany). The insect’s nymph has 5 instars, instar 1–2, 3–4, and 5 are categorised into small, medium and large nymphs, respectively. The nymphs can be distinguished by their sizes: instar 1, instar 2, instar 3, instar 4, and instar 5 are approximately 1.5 mm, 2.0 mm, 3 mm, 5 mm, and 7 mm in body length, respectively (Rahayu *et al.* 2015). Besides that, the nymphal stadia can also be used to estimate the instars of the nymphs: instar 1, instar 2, instar 3, instar 4, and instar 5 last 2–5 days, 2–3 days, 2–3 days, 2–5 days, and 4–5 days, respectively (Lestari 2014).

### Data Analysis

Data collected were analysed using ANOVA. A significant difference among treatments was detected then the treatment means were separated using a Duncan’s multiple range test at 0.05.

## RESULTS AND DISCUSSION

During the vacuuming process, most insects present on the sample plants were collected. Other than the rice grain bug, several insect pests of rice such as rice brown planthoppers (*Nilaparvata lugens* Stal), rice green leafhoppers (*Nephotettix* spp.), and rice bug (*Leptocorisa* sp.) were present in the trial plots. In addition, several predatory insects such as coccinellids and lycosids; parasitoids such as braconids, and ichneumonids were also collected from the sample plants. Rice grain bug collected in this research were both nymphs and imagoes. Adult rice grain bug had a body length of 6.5–7.5 mm; almost their entire abdomen was covered by very hard, dark brown wings. When disturbed, imago would fly away or drop off to the ground. The nymph specimens were categorised based on their size: large and medium nymphs were about 7 and 3–5 mm in length. Medium nymphs were very active and travel by means of walking. Small nymphs were those that measured smaller than 2 mm and travel by means of walking and can drop off the plant when disturbed. Based on the body size, the small nymphs were instars 1 and 2, the medium nymphs were instars 3 and 4, and the large nymphs were instar 5 (Lestari 2014; Rahayu *et al*. 2015).

There was a general trend that the greatest population of *P. pallicornis* was found in weedy field, followed by the weed-free field, and then the paddy dykes (Tables 1, 2 and 3). On the observations of 85 days and 105 days after transplanting, total number of insects, number of imago, number of large, medium, and small nymphs in weedy field was significantly higher than the population in paddy dykes but not significantly different from the population in weed-free field (Tables 1 and 2). These higher numbers of adults and large nymphs in weedy fields appear to be caused by two factors. The first factor is that rice grain bug invasions were probably initiated in weedy fields, and the second factor is the availability of food and environmental protection that a weedy field provides. Rauf (1996) noted that the rate of insect colonisation is influenced by the time of invasion and the availability of food and protection.

The high density of vegetation in paddy dykes perhaps provided sufficient environmental protection for the rice grain bug, but the relative scarcity of food sources caused much lower population numbers in dykes compared to paddy fields. Conversely, weed-free fields had an abundance of food for the black rice bug, but perhaps provided less protection to the insect. This is in accordance to previous observations (Patta *et al.* 2009) concluded that rice grain bug have a tendency to hide or take cover in the stem of the paddy plant during the day, only becoming active to feed on the plant sap in the early evening.

On the observation of 115 days after transplanting (1 day after harvest), although the bug population in weedy field was still the highest among the treatments, the number of insects in weed-free field decreased and in the dykes increased. Therefore, the numbers of insects in weed-free field was not significantly different from those in the dykes (Table 3). However, the numbers of adults were not significantly different amongst the treatments. This appeared to be caused by the disruptive effects of harvesting and the capability of imago to fly or migrate to other locations. These results indicated that paddy dykes serve as sinks for rice grain bug after crop harvest. In the rice paddy fields*, Echinichloa* spp., *Cyperus* spp., *Eleusine indica*, *Vigna sesquipedales*, and *Paspalum conjugatum* are weed species reportedly as alternative hosts of *P. pallicornis* (Conado 2013). The most abundant weeds in the study area were *Echinichloa* spp. and *Cyperus* spp. The weeds could serve as temporary hosts for the bug when rice is not available; however, when rice plants are available they will move to the main crops. Therefore, keeping weed populations low in rice production system, not only within the crop fields but also in the irrigation ditches and fence rows, is a recommended practice because those areas can become sources of insect infestation (Capinera 2001; Metcalf & Metcalf 1993).

Total population average of rice grain bug was significantly higher in weedy paddy fields after the harvest, apparently due to the higher numbers of medium and small nymph populations compared to weed-free fields and paddy dykes. Such high medium and small nymph populations in weedy paddy fields appear to be caused by high existing populations and the inability for the rice grain bug to swiftly migrate to other habitats at that stage of development. These may then become a rice grain bug population source that attack crops in the following season. The results of this research show very high rice grain bug populations in weedy paddy plots, thus weed control is necessary to suppress the bug populations. Similarly, paddy dykes serve as sinks for migrant rice grain bug and must be properly handled. If weeds or volunteer plants are not timely controlled, they can serve as sources for large numbers of insect pests and plant viruses transmitted by insect vectors that can migrate to other crops both in current and next planting seasons (Palumbo 2013).

In summary, during planting season rice grain bug population was higher in weedy field compared to the weed-free field and paddy dykes. However, after harvest the population decreased in weed-free field, remained about the same in weedy field, and increased in dykes. The results suggested that weeds play important roles in regulating the pest population, therefore, weeds both in the field and dykes must be properly control to prevent high investation of the insect pest. However, weed plants can also serve as a refuge for other arhtropods that are natural enemies of insect pests, hence the presence of weeds in the field is beneficial to agricultural operation. Thus, future study should focus on the balance between controlling the bug by reducing the population of the weeds and maintaing the weeds for conserving the natural enemies.

## Figures and Tables

**Table 1:** Average number of rice grain bug per two hills at plant age of 85 days after transplanting.

Treatment	Average number per two hills				

	Imago	Big nymph	Medium nymph	Small nymph	Total
Weed-free field	0.29ab	0.42ab	0.54a	0.50a	1.75ab
Weedy field	0.92b	0.81b	1.00a	0.86a	3.53b
Paddy dykes*	0.06a	0.17a	0.08a	0.00a	0.31a

Numbers followed by the same letter at the same column were not significantly different (P = 0.05, Duncan’s multiple range test).

* Numbers of insect per 0.3 m^2^

**Table 2:** Average number of rice grain bug per two hills at plant age of 105 days after transplanting.

Treatment	Average number per two hills				

	Imago	Big nymph	Medium nymph	Small nymph	Total
Weed-free field	0.67ab	0.75ab	0.64ab	0.47ab	2.53ab
Weedy field	1.75b	1.36b	0.97b	1.44b	5.53b
Paddy dykes*	0.06a	0.17a	0.00a	0.00a	0.11a

Numbers followed by the same letter at the same column were not significantly different (P = 0.05, Duncan’s multiple range test).

* Numbers of insect per 0.3 m^2^

**Table 3:** Average number of rice grain bug per two hills at plant age of 115 days after transplanting.

Treatment	Average number per two hills				

	Imago	Big nymph	Medium nymph	Small nymph	Total
Weed-free field	0.21a	0.29a	0.17a	0.04a	0.17a
Weedy field	0.88a	1.17b	0.88b	0.83b	3.75b
Paddy dykes*	0.17a	0.19a	0.06a	0.08a	0.50a

Numbers followed by the same letter at the same column were not significantly different (P = 0.05, Duncan’s multiple range test).

* Numbers of insect per 0.3 m^2^
